# Prevalence of stress and its relevance on psychological well-being of the teaching profession:  A scoping review

**DOI:** 10.12688/f1000research.131894.1

**Published:** 2023-04-20

**Authors:** Shilpa Badrinath Bidi, Varalakshmi Alapati, Venisha Jenifer Dmello, Edwin Weesie, Mathew Thomas Gil, Sandeep S Shenoy, Simmy Kurian, Ambigai Rajendran

**Affiliations:** 1 Department of Commerce, Manipal Academy of Higher Education, Manipal, Karnataka, 576104, India; 2 Lectorate Finance Economic Innovation, University of Applied Sciences,, Heidelberglaan 15, 3584 CS, Utrecht, Heidelberglaan, 85029 – 3508, Netherlands Antilles; 3 Management studies, Jain (Deemed-to-be University), Kochi campus, Kerala, India

**Keywords:** stress factors, stressors, well-being, psychological well-being, teachers, educator, interventions.

## Abstract

**Background**: Well-being among teachers contributes toward quality work and better student support. Teachers’ well-being persists to be a concern in school settings; there is a lacuna in understanding the concept of well-being among them. This scoping review identifies the stress factors and map their association with the psychological well-being of teachers employed in schools. Additionally, to identify the methodology and the interventions used in reducing teachers' stress and their relevance on their psychological well-being.

**Methods**: First, Pubmed, Web of Science, and Scopus databases were searched for eligible studies with MeSH terms for stress factors, well-being, and teachers from 2010 to 2022. Identified studies were screened thoroughly and excluded or included based on prior established criteria. Data from the included studies were extracted and summarized according to the study protocol.

**Results**: Among the 60 studies that met our inclusion criteria, the majority were quantitative, with cross-sectional studies. Several studies focused on emotional exhaustion, depersonalization, and diminished personal accomplishment aspect among teachers. Almost half of the included studies focused on organizational and social pressures such as administration workload, classroom management issues, lack of supervisor and team support, students’ behaviour, and pressure from parents. The most used interventions to overcome stress were coping strategies and mindfulness training intervention tools.

**Conclusions**: The findings from the current scoping review will reveal the different stressors which impact psychological well-being. Focus on the most used interventions to overcome stress among schoolteachers. This will also provide recommendations to regulators and management to identify the factors causing stress among teachers and their relevance to their psychological well-being, overcome employee turnover and absenteeism issues. Also, different alternatives available to reduce the stress may benefit the stakeholders and policymakers to confirm a suitable intervention that will benefit the teaching profession.

## Introduction

The process of instruction known as education aims to help people develop their knowledge, attitude, and character for giving them the tools they need to live purposeful lives. Our educational system's most critical connection is the teacher. The effectiveness of the teachers is a major factor in the outcome of any educational program. Well-being as a topic has been investigated since the last two decades ( Schellenbaum *et al*., 2002; Schaufeli & Bakker, 2004; Pritchard *et al*., 2019) and has become an increasingly popular topic in academic and business settings, with organisations attempting to discover what makes working environments engaging and motivating. The key objective of developing organisational understanding and offering development to the workforce has become a major element. Thereby the responsibility of the future generation, by and large, is bestowed upon the educational institution. There are claims stating that education is a process that directs and manages the behaviour of individuals of all ages irrespective of time and place and really not restricted to schools or educational institutions ( Howard, 1993). Even today, there is an existence of strong culture that believes educational systems are accountable for the civilization of society and for the development of individuals ( Turkkahraman, 2015). Thus, educational institutions have become vital and indispensable work setup in the contemporary situation. Teachers are considered as a driving force of learning organizations to foster learning than ever before, and thus they become ‘knowledge workers’ to effectively deal with the rapidly changing environment for improving learning outcomes. The welfare and well-being of such knowledge workers require primary focus from the regulators and managements.

Studies conducted in various countries shows high stress levels and mental disorder among teachers ( Wu *et al.*, 2006; Yu *et al*., 2014). Approximately 60% of teachers reported they are stressed ( Gomez, 2022) globally. Professional burnout is also caused by continuous teacher stress and inadequate coping mechanisms ( Maslach *et al*., 2001). Research has revealed that over a one third of education professionals are expected to leave their job by 2020, highlighting the importance of teacher mental health and the need to address this crisis ( Hazlegreaves, 2020).

Well-being among teachers contributes towards quality work, self-efficacy in the classroom and better student support. In many countries, psychological well-being among teachers has become a growing concern ( Seibt *et al*., 2013; Alhija, 2015; Mahfar *et al*., 2018; Anderson *et al.* 2022). Nonetheless, most school-based research has focused on pupils rather than teachers' health and well-being ( Moy *et al*., 2014). Teachers plays an integral role in shaping the future generation in their provided workspace and thus in turn it affects the well-being of the society directly. Hence, our study helps to identify the factors causing stress among teachers and its relevance on their psychological well-being. Also, different measures taken to reduce the stress, which intern may also benefit the stake holders policy makers to adopt the suitable interventions which will benefit the teaching profession.

The objective of our review is to scope the extent and nature of the articles investigating stress factors on psychological well-being among teachers. We aim to identify gaps in the literature and use the results of our search to inform decisions about directions for future research. A preliminary review on this topic was conducted in April 2022 using JBI evidence synthesis and Open Science Framework. We were unable to find any planned or existing scoping reviews with the same focus as ours.

## Methods

### Design

The main objective of the study is (a) to explore the stressors influencing the psychological well-being of teachers. (b) to map the measures/interventions which helped in managing the stress. To attain the objectives of the study scoping review was conducted in 2022. The methodological framework adhered to the guidelines proposed by Arksey and O’Malley (2005) and was developed further by Levac and his colleagues ( Levac *et al*., 2010). Besides the Joanna Briggs Institute (JBI) guidelines were followed with the following methodological steps: (a) Identification of research question; (b) Identification of studies which are relevant; (c) Selection of studies; (d) Charting of data; (e) Collation of results, summary and report. The scoping review was not entered into the PROSPERO database in accordance with the study’s design.

(a) To explore the stressors influencing the psychological well-being of teachers.
(b) To map the measures/interventions which helped in managing the stress.

### Research questions

A scoping review is a predominantly adopted technique for appraising and synthesizing literature evidence which gives the broader prospect and the extent of the research activity in terms of the topic proposed, mapping the literature gaps and finally provide future direction to the researchers. Based on the objectives, the review addresses the following research questions:

a. What are the descriptive findings of the selected studies?
b. What are the core stress factors influencing psychological well-being of schoolteachers?
c. What are the measures/interventions adopted in managing stress among schoolteachers?

### Search strategy

The preliminary search for the study was conducted in the National library of medicine database to define the search strategy and to identify key terms. Once key terms and MeSH terms were identified, three databases (Scopus, Web of Science and PubMed) were used to collect the relevant articles. These databases were considered as a majority of the content published is indexed in reputed journals. The search strategy was performed using various keywords, phrases and Boolean operators based on the combination of the following: stress OR burnout OR cynicism AND perceived stress OR stress factors OR personal stress OR work-related stress OR job-related stress AND well-being OR wellbeing OR psychological well-being OR psychological health OR mental well-being AND teachers OR instructors OR educators OR academicians OR facilitators OR school teachers OR elementary schools. In addition to the electronic search, a manual search using the snowball technique and reference lists was conducted of studies that qualify. The search terms were restricted to title, abstract and keywords in Scopus and PubMed, in Web of Science title, abstract, author keyword and keyword plus.

### Inclusion and exclusion criteria

Studies were considered and included if they met the following inclusion criteria:

**Table 1.** Inclusion and exclusion criteria.

Criterion	Inclusion	Exclusion
Time	2010-2022	Articles or book chapters outside these dates.
Language	English	Foreign language articles or book chapters.
Type of article	• Articles published in peer-reviewed journals. • Review articles bibliography was checked as additional search. • Articles that adopted Quantitative, Qualitative and a Mixed approach	• Articles and book chapters that were not peer reviewed. • Review articles. • All reports, conceptual papers, lectures and conference proceedings.
Topic focus	• Articles that examined: stress factors measures/interventions to overcome stress in alliance with psychological well-being	• Articles that examined stress factors in alliance with occupational well-being. • Measures/interventions not emphasising on overcoming stress.
Population and sample	School teachers who teach primary, secondary and high school section. Both male and female	School teachers who teach for kindergarten, higher education, Special education teachers and Language teachers

The central focus of the study was to understand the impact of stress on well-being mainly due to the burgeoning studies on teachers’ well-being in the past two decades. Research articles mainly focusing on evaluating stress among SEN, language teachers or any other teachers were not considered as it was not in the scope of the study. However, this suggestion is addressed by proposing to future researchers to conduct a comparative review or study by considering the above-mentioned segments.

All eligible studies were further screened by two researchers independently with the title and abstract in Rayyan software by excluding duplicates and considered the full text for data extraction. Third researcher was involved to resolve discrepancy through discussion. The reference lists of systematic reviews were examined to recognise possible eligible studies.

**Figure 1.**
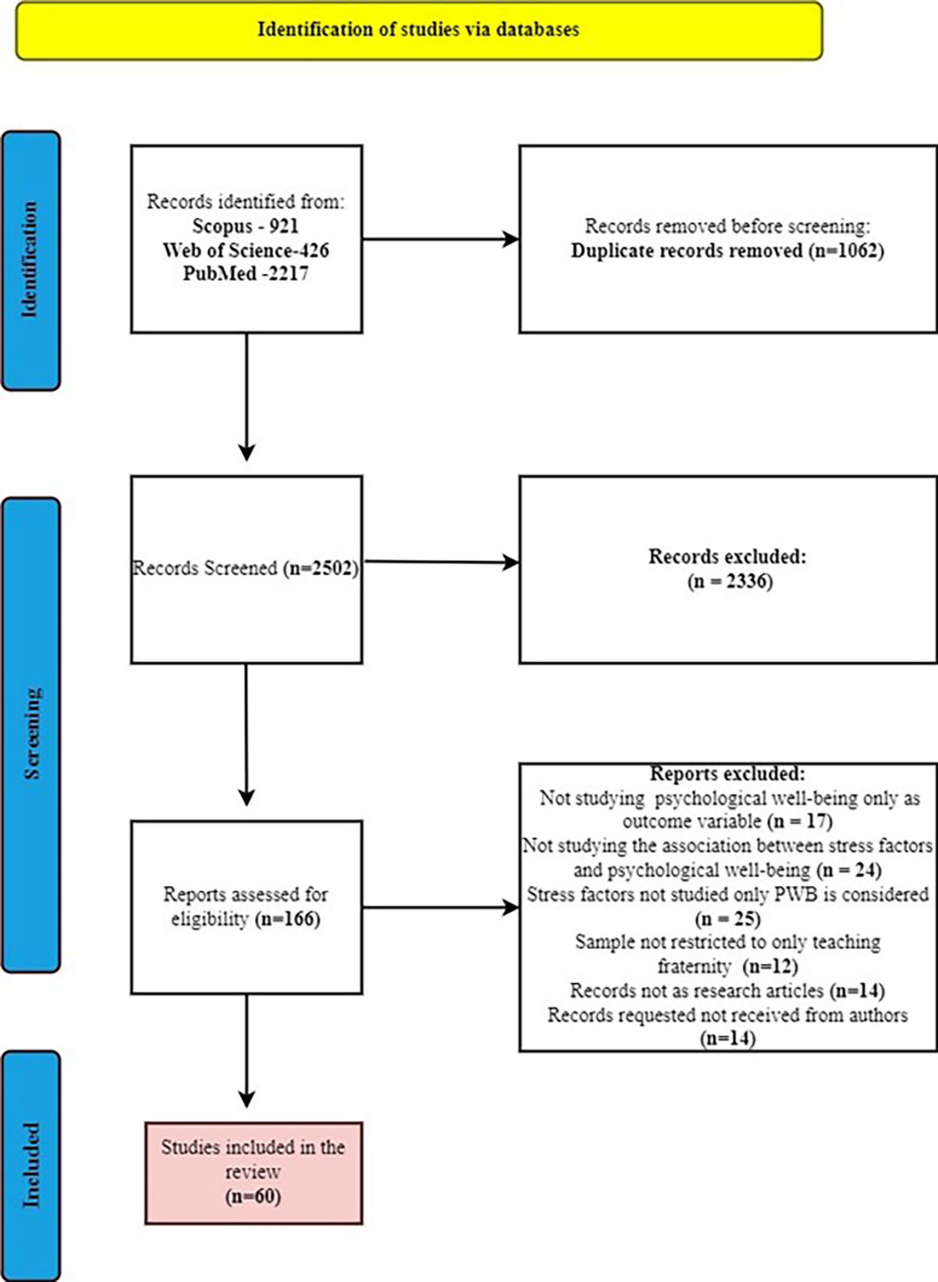
PRISMA flow chart.

### Inclusion decision based on inclusion and exclusion criteria

The search resulted initially in total of 3564 hits, duplicates were removed in Microsoft Excel which resulted in up to 2502 articles. All the refined articles were added to Raayan software for title and abstract screening. Two independent researchers screened and in any case of any discrepancies, was solved by discussion with the third researcher ( Brereton *et al*., 2007). With the reasons records of other professional samples (1431), records with outcome other than psychological well-being (862), records with review papers (15), records with book chapters (10) and records with foreign language (18) were excluded which resulted to 166 articles were considered for full paper screening. Further, among 166 articles by full paper screening we excluded articles with reasons such as studies not considered psychological well-being only as outcome variable (17), articles not studying the association between stress and psychological well-being (24), articles studying stress factors associated with variables other than psychological well-being (25), articles not solely focused on the teaching profession (12), records with book chapters (14), records requested to authors but not received (14). Finally, 60 articles were considered for the study.

### Charting the data

A piloted and standardised data extraction form was used by two reviewers to independently collect the data and third as a checker ( Brereton *et al*., 2007). Finally 60 selected articles were reviewed separately by two authors in order to analyse and record the data. Each article was summarized in Microsoft Excel carefully, by recording following main elements: ‘Bibliographic information such as, extent i.e., year of publication, title, author, country, objective of the study, methodology, sample size, data analysis, findings, stress factors, measures to overcome stress, future scope and limitations. During the process, two researchers compared their Excel forms and discussed if any disagreements in weekly meetings. A third researcher cross checked the information.

## Results

A summary of the recognised studies is provided in this section as listed in Appendix A. First, we report descriptive statistics related to publication date, research context, geographical setting and theories adopted by the researchers. Next, we categorize different stress factors among the teaching profession and measures/interventions techniques employed to reduce the impact of stress.

### Findings

***What are the descriptive findings of the selected studies?***

### Descriptive findings

*Publication period*

**Figure 2.**
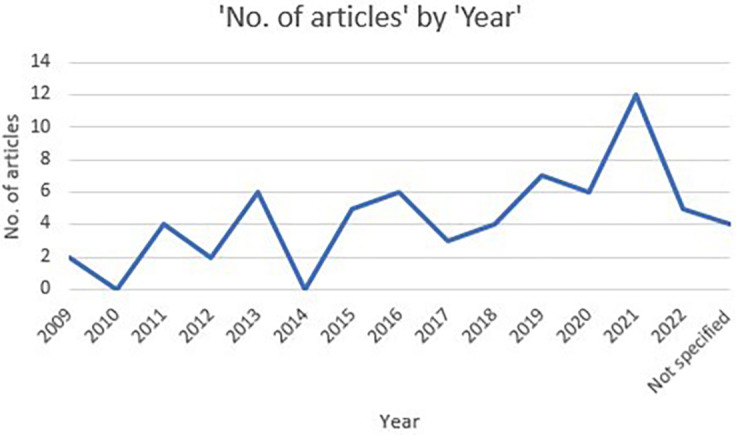
Publication trend of included articles.

Figure 2 shows the annual distribution of selected articles. As seen, the publication of articles on stress factors aligned with psychological well-being among schoolteachers are very few in 2010, a moderate increase can be noticed in 2013. In 2014, there was no studies recorded. From 2016 to 2020 there was a substantial increase in studies. Maximum records were identified recently in the year 2021 i.e., post covid period.

*The geographic distribution*

**Figure 3.**
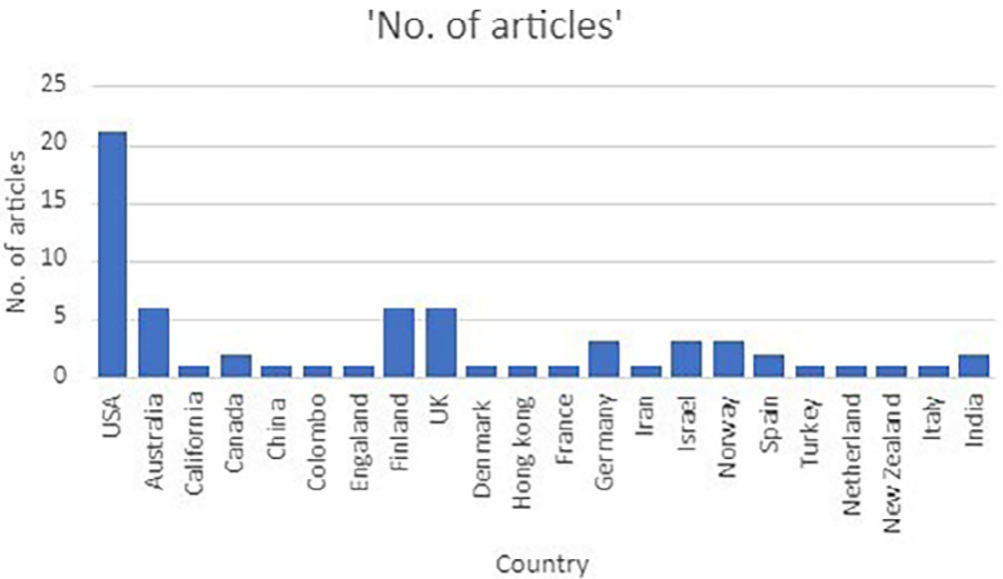
Geographical distribution of included articles.

Figure 3 shows the geographic distribution of the selected articles. It can be seen that stress factors and psychological well-being has been studied most in USA (N = 21). Followed by Australia (N = 6), Finland (N = 6), UK (N = 6), Israel (N = 4), Norway (N = 4), Canada and Spain (N = 3). Regarding Colombo, Iran, Italy, India, Turkey, China two each were found.

*Research methodology*

There is tendency to use quantitative research methods in studying stress factors associated with psychological well-being among schoolteachers. In total, 37 publications employed quantitative research methods, whereas 13 publications employed qualitative, and 10 publications employed mixed methods respectively. Most of the studies adopted survey and randomized control trail (RCT) methods to collect the data based on statistical analysis, used SPSS and structural equational modelling (SEM), correlation/regression analysis, ANOVA/ANCOVA and t-test to test the hypothesis. Whereas qualitative method adopted in-depth interviews, semi structured interviews and focus group interviews. Mixed methods adopted both survey and interviews for the better understanding of the research problem. Table 2 presents the main research methodologies adopted in the selected publications.

**Table 2.** Research methods adopted in the selected articles.

Research approach	Research method	No. of articles
Quantitative approach	Survey/Randomized control trail	37
Qualitative approach	In-depth interviews, semi structured interviews and focus group interviews	13
Mixed approach	Both survey and interviews	10

*Theoretical perspectives*

Table 3 presents the theory which were adopted by authors among the selected studies. Job Demand and Resource model theory ( Demerouti *et al*., 2001) is the maximum adopted theory by the researchers followed by self-determination theory, teacher performance motivation theory. PERMA theory coined by Seligman (2018) has captured researcher's attention in recent years. Along with these social cognitive theories, theory of planned behaviour, green glass theory, psychological theory of well-being and many more were used.

**Table 3.** Theoretical underpinnings of the selected studies.

Theory	No. of articles
JDR – Theory	4
Self-determination Theory	3
Teacher Performance Motivation Theory	2
PERMA Theory	2
Social cognitive Theory	1
Theory of Planned behaviour	1
Greenglass’ Theory	1
Psychological theory of well-being	1
Qualitative Grounded Theory	1
Conservation of resources	1
Transaction and coping Theory	1
Transactional stress Theory	1
Social–psychological Theory	1
Theory of Preventive Stress Management	1
Others	45

***What are the core stress factors influencing on psychological well-being of schoolteachers?***

A number of studies have proved teachers are prone to higher level of stress ( Boyle *et al*., 1995; Burke *et al*., 1996; Bhuin, 2017) exposure to higher stress levels results in adverse consequences on health and well-being which in turn leads to poor performance, low work engagement, negative impact on the quality of student learning/education, employee turnover rates and absenteeism ( Kyriacou, 2011; Hoglund *et al.* 2015; Von der Embse *et al*., 2016). Various researchers have identified different stress factors that influence the psychological well-being of the teaching profession. Although extensive research has been carried out on identifying the stress factors very few researchers have conducted a scoping review to explore the key stressors influencing psychological well-being. In order to address the research question, factors causing stress were coded and analysed using a thematic analysis approach. The main themes ( Figure 5) on the stress factors were arrived on the basis of Bronfenbrenner’s ecological systems framework ( Bronfenbrenner, 1992). It has emerged as an effective framework as it captures a wide range of stress factors ranging from micro and macro systems ( Farley & Chamberlain, 2021) The stress factors were categorised into intrapersonal, systematic, relational and organisational stressors.

***Systematic stress factors***

The systematic stressors theme comprised of national and state level policies that manage the education system operating. Holistic changes in the policies impose burdens on teachers to professionally equip with the required alterations. The review identified approximate 7% of the factors were contributed from systematic stressors like coping with changes in the policies ( Yerdelen *et al*., 2016), Inefficient working policies ( Skaalvik & Skaalvik, 2018), Constant changes in policies ( Alvarado & Bretones, 2018; Farley & Chamberlain, 2021), Education reforms ( Alloh *et al*., 2019; Hepburn *et al*., 2021c), National & State level policies ( Schnaider-Levi *et al*., 2017; Carroll *et al*., 2020; Gearhart *et al*., 2022).

***Organisational stress factors***

**Figure 4.**
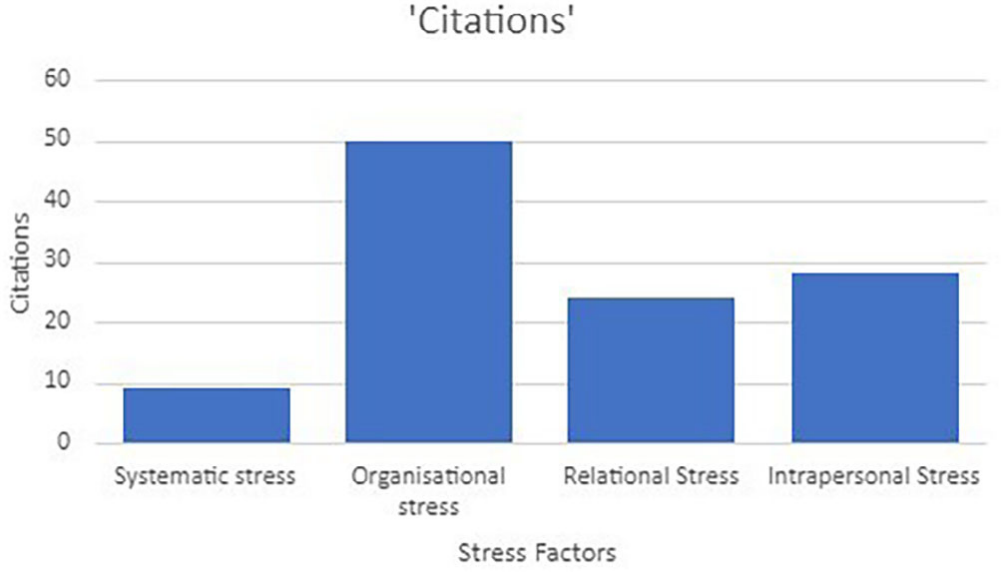
Stress factors classification.

The organisational stress theme comprised of significant factors in the work place which had an influence on psychological well-being. Within this theme around 48% of the factors contributed to various categories of stress. They were multi-tasking ( Gold *et al*., 2009), workload ( Gold *et al*., 2009; Simbula *et al*., 2012; Vazi *et al*., 2013; Vesely *et al*., 2013; Bermejo-Toro *et al*., 2015; Desrumaux *et al.*, 2015; Yerdelen *et al*., 2016; Helms-Lorenz & Maulana, 2016; Skaalvik & Skaalvik, 2017; Alvarado & Bretones, 2018; Alloh *et al*., 2019; Braun *et al*., 2019; Soykan *et al*., 2019; Brady & Wilson, 2020; Carroll *et al*., 2020; Farley & Chamberlain, 2021; Hepburn *et al*., 2021a; Ibrahim *et al*., 2021; Tebben *et al*., 2021; Billett *et al*., 2022), lack of professional recognition and appreciation, administration overload, role conflict, poor working condition, time pressure, lack of recourses and technology, appeared as organisational factors which contributed to teachers' stress. Workload was the prominent category which was recorded in most of the studies to be the key factor for emotional exhaustion among teachers ( Bower & Carroll, 2017) and burn out ( Chang, 2009). Often inseparably connected with workload was due to increasing work demands from leadership team, extensive student performance and short deadlines. Factors such as lack of autonomy, job insecurity, technological challenges also were the reasons for creating stress among teachers ( Figure 4).

***Relational stress factors***

Within the theme of relational factors, there were many categories which contributed up to 21% of the stress factors. Authors mentioned, parent demands and expectations ( Carroll *et al*., 2020), low level of depersonalization ( Luk *et al*., 2010; Brown & Roloff, 2011; Ross *et al*., 2011; Klassen *et al*., 2012; Alloh *et al*., 2019), Student behaviour & massive class strength, interpersonal relations with colleagues and students, maintaining discipline and teaching preparation ( Yerdelen *et al*., 2016) were the contributing factors under this theme.

***Intrapersonal Stress factors***

The theme intrapersonal stress comprised of factors that were not related to work directly. Ibrahim *et al*. (2021) and Gearhart *et al*. (2022) found emotional exhaustion and lack of coping abilities with challenges ( Alhija, 2015; Mahipalan & Sheena, 2019; Carroll *et al*., 2020; Hepburn *et al*., 2021c) as the prime factors for intrapersonal stress. Followed by inequity Work/family conflict ( Jerrim & Sims, 2021; Billett *et al*., 2022; Gearhart *et al*., 2022), Low self-efficacy level ( Vesely *et al*., 2013; Bermejo-Toro *et al*., 2015; Prilleltensky *et al*., 2016), Lack of experience ( Ibrahim *et al*., 2021), Low self-efficacy level ( Schnaider-Levi *et al*., 2017; Braun *et al*., 2019).

**Figure 5.**
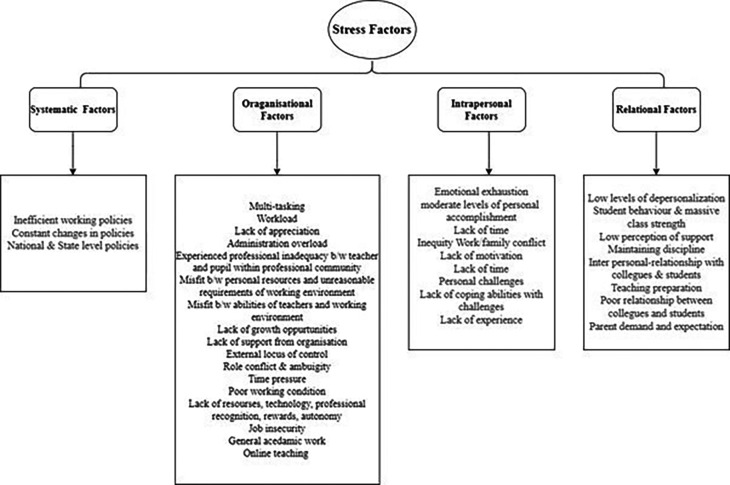
Overview of stress factors identified on the basis of Bronfenbrenner's ecological systems framework. Source: Authors own.

***What are the measures/interventions adopted in managing stress among schoolteachers*?**

The number of therapies, interventions and measures have increased in lowering employee stress in recent years. Although ample research has identified different ways to manage stress among teachers very few studies have carried out scoping reviews to explore intervention or measures in align with psychological well-being. To address the research question, three different ways of measures/interventions were coded and analysed using a thematic analysis approach. There were five themes identified from the selected studies. They are mindfulness-based intervention, improved social support, coping mechanisms, improved managerial support and others.

***Mindfulness-based intervention***

Mindfulness can be defined as “a state of being attentive to and aware of what is taking place in the present” ( Brown & Ryan 2003, p. 822). Under this theme, Cultivating Awareness and Resilience for Teachers (CARE) ( Roeser *et al*., 2012; Jennings *et al*., 2013), Stress management and relaxation techniques (SMART) ( Roeser *et al*., 2013; Jennings *et al*., 2013), Co-ordinated Anxiety Learning and Management program (CALM) ( Jennings *et al*., 2013; Jennings *et al*., 2019; Hepburn *et al*., 2021a), Inner Resilience Mindful based training ( Frank *et al*., 2013; Beshai *et al*., 2015; Dave *et al.*, 2020; Bonde *et al*., 2022), Emotional Intelligence skill training ( Vesely *et al*., 2013; Schoeps *et al*., 2019), Mindfulness stress reduction practice such as walking, stretching, body awareness reflection, deep breathing ( Jennings *et al*., 2013; Hepburn *et al*., 2021a), RCT through training sessions ( Siu *et al*., 2014), training sessions on gratitude, happiness day (an activity which consist of planning and carrying out of positive emotions for example activities such as visit to the parlour for self-care, an outing or musical experiences.), savouring experience (“savoring makes a decisive contribution to experiencing and intensifying positive emotions” Bryant *et al*. (2011)., letter ( Rahm & Heise, 2019), social-emotional skill training ( Tarrasch *et al*., 2020), training & classes to promote well-being ( Tebben *et al*., 2021), Mentalising ( Schwarzer *et al*., 2021), intervention - brief PPIs (BPPIs)- ( Shankland & Rosset, 2016), Mindfulness training ( Bonde *et al*., 2022) were identified. Recently yoga based intervention ( Harris *et al*., 2015; Telles *et al*., 2018; Dyer *et al*., 2020; Hepburn *et al*., 2021c) was the most commonly adopted intervention to overcome stress.

***Improved social support***

Under this theme, the measures which are adopted through social support such as colleagues, students and family were recorded. They are: building of inter-personal relationships ( Ibrahim *et al*., 2021), Positive interactions with students ( Ross *et al*., 2011), collaboration between teachers and colleagues ( Helms-Lorenz & Maulana, 2016; Round *et al*., 2022), Positive social climate ( Aldrup *et al*., 2017), interpersonal support environment ( Soykan *et al*., 2019), mentor and coach collaborative and reflective relationship ( Hollweck, 2019; Carroll *et al*., 2020), Community bonding ( Gearhart *et al*., 2022), Positive and supportive relations with colleagues and parents and collective culture ( Skaalvik & Skaalvik, 2017) were identified under this theme.

***Coping mechanisms***

Coping mechanisms which were employed to overcome stress has been recorded under this theme. Jennings found that emotional skill instruction helped in managing the prevailing stress ( Jennings *et al*., 2013). School enculturation was the technique used by Helms ( Helms-Lorenz & Maulana, 2016). Followed by an improvement in communication skills ( Carroll *et al*., 2020), sessions on stress management techniques ( Li *et al*., 2016), cognitive and behavioural coping ( Bermejo-Toro *et al*., 2015; Prilleltensky *et al*., 2016; Aulén *et al*., 2021; Turner *et al.*, 2021; Ciuhan *et al*., 2022) feeling valued and fringe benefits ( Gearhart *et al*., 2022), workplace change ( Carroll *et al*., 2020) and health promotion ( Vazi *et al*., 2013) were gathered from the selected studies.

***Improved managerial support***

**Figure 6.**
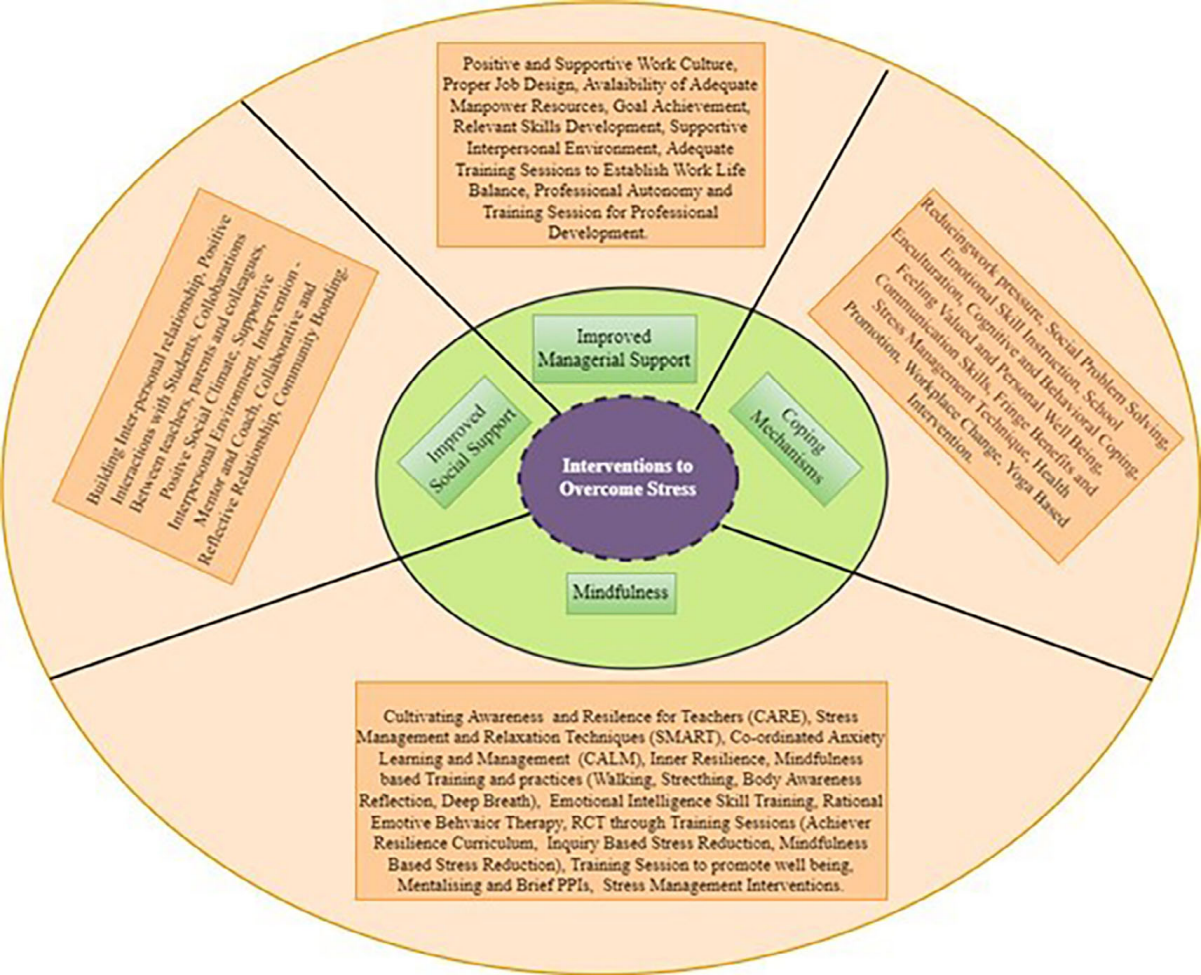
Intervention classification. Source: Authors own.

This theme comprises of the measures which was adopted by management to reduce stress mong teachers. Many researchers found building of interpersonal relationships among colleagues and management helped in reducing stress ( Wu *et al.*, 2006; Luk *et al*., 2010; Ibrahim *et al*., 2021; Prilleltensky *et al*., 2016) further, positive interaction with students ( Ross *et al*., 2011), Positive & supportive relations with colleagues and parents, collective culture ( Skaalvik & Skaalvik, 2017) and community bonding ( Gearhart *et al*., 2022) were the thoughts adopted by management in lowering stress among teachers ( Figure 6).

## Discussion

Houdmont, Cox, and Griffiths (2010) argued, the assessment of interventions related to work is challenging; framing, conceptualising and methodology regarding interventions that supports to overcome the problem is still an open debate. "The decision about which type of intervention to use should be based on a thorough assessment of the specific situation rather than some general principles” ( Briner and Reynolds 1999, p. 658). Interventions cannot be carried out purely based on broad theoretical ideas; they must be tailored to the unique organizational context in which they are implemented. Ecologically sound and measurable interventions are required. Taking this viewpoint into account, the goal of this study was to a set out with the main aim of identifying the stress factors and assessing the significance of interventions/measures to reduce stress for the psychological well-being among schoolteachers. Additionally, the study also aimed to ascertain the overview of methodologies, geographical distributions, theoretical underpinnings and publication period with the said topic. Therefore, to address the research questions, scoping review methodology adhering to JBI guidelines was adopted.

### Publication period

The results of the review showed that the articles on stress aligned with psychological well-being among schoolteachers were few in the year 2010, gradual increase was recorded between 2013 to 2020 and exponential growth was recorded in the year 2021. The significant growth in the articles is associated with the outbreak of the global pandemic, called for incorporating the additional factors such as technological challenge ( Farley & Chamberlain, 2021; Ibrahim *et al*., 2021), lack of work life balance ( Billett *et al*., 2022; Gearhart *et al*., 2022), student behaviour management ( Tebben *et al*., 2021; Hepburn *et al*., 2021a) and online teaching ( Billett *et al*., 2022) along with the prevailing factors that elevated stress among the teaching profession.

### The geographic distribution

The geographical distribution of the study shows that most studies emerged from US (n = 16) followed by UK, Finland and Australia (n = 5). The geographical distribution of the selected articles in the review demonstrates stress and interventions as a measure to overcome stress has captured global attention. However, the review highlights a lacuna of studies emerging from developing countries such as India, Iran and Turkey.

### Research methods adopted in selected articles

60 studies ranging from the year 2010 to 2022 were selected that fulfilled inclusion and exclusion criteria set out for the study. Among them, 37 studies adopted quantitative method, 13 studies adopted qualitative method and 10 articles adopted mixed methodology. It is apparent from the review that, quantitative research design was extensively used method by the researchers to examine the phenomena.

### Theoretical perspective

An application of a strong theoretical background enhances the outcome of the study in terms of proposing theoretical and practical implications. Through review it was observed that researchers have adopted various psychological, psychosocial, and behavioural theories. JD R theory ( Demerouti *et al*., 2001) strongly guided the stress and well-being literature. However, these results are not surprising as the existing literature has confirmed the application of JD R models from past two decades. Furthermore, positive emotion, engagement, positive relationships, meaning and accomplishment which is termed as “PERMA” ( Seligman 2011) was used as a core theory to measure the psychological well-being. This model has exhibited a great heuristic power in stimulating enormous studies due to integration of various theories in positive psychology.

### Stress & intervention

Data from teaching and learning survey reports confirmed the existence of high level of stress in teaching profession ( Carroll *et al*., 2022). High level of stress impacts the job performance, mental and physical health. Another study by ( Della Valle *et al*., 2020) expressed chronic stress among employees results in huge economic burden due to low productivity and engagement. The current study emphasised on identifying the drivers of the stress. The stressors were classified into intrapersonal, systematic, relational and organisational stressors. Among the studies included in the review it could be observed that workload (22 studies), time pressure and gap between job demand and availability of resources (6 studies), administration overload (4 studies), role conflict and ambiguity (4 studies), poor working condition (4 studies), job insecurity (3 studies), lack of administrative support (2 studies), and lack of appreciation (1 study) were the commonly studied organisational stress factors. Further, massive class strength, poor relationship between teacher, student, and colleagues (13 studies), managing supervisors (9 studies), low levels of depersonalization (5 studies) and managing parents demand and expectations (2 studies) were highlighted under relational stress factors. Additionally, intrapersonal factors such as Low self-efficacy level (10 studies), emotional exhaustion (8 studies), Inequity Work/family conflict (7 studies), moderate levels of personal accomplishment (4 studies), Lack of coping abilities with challenges (3 studies), Lack of experience (3 studies), Lack of time (2 studies), Lack of motivation (2 studies) Personal challenges (1 study). Among the mentioned factors “workload”, poor relation between students, teachers and colleagues”, low self-efficacy and emotional exhaustion were frequently studied by the researchers. There were relatively lower studies focusing on policy related factors. Overall researchers empirically validated that excessive stress levels caused unpleasant outcomes such as low productivity, dissatisfaction, job turnover, low job performance, increased anxiety levels, sleep disorder, insomnia and chronic health problems. Moreover, the negative consequences of stress did not restrict only to the facilitators but also affected the students' academic performance. Stress management interventions act as an antidote for overcoming the negative consequence of stressors. Various strategies have been developed by researchers to reduce the impact of stress on the well-being of the desired population. Stress management interventions at the workplace emphasise on designing ameliorative tactics to prevent and restore the well-being of employees through promotive wellness programs. Therefore, interventions identified through this scoping review were aligned with the stress factors which were deployed to overcome stress among the teaching profession.

The most common intervention adopted to overcome organisational, intrapersonal and relational stressors was mindfulness (30 studies). It mainly comprised of CARE, CALM and SMART programmes. These programmes were conducted based on randomised control trials. Study by ( Roeser *et al*., 2012; Jennings *et al*., 2013, 2019; Carroll *et al*., 2020) examined the effectiveness of CARE program package such as emotional skills instructions, mindful awareness, caring and compassion practices. It resulted in proximal outcomes such as emotional stability, decrease in psychological and physical distress. The cohort which underwent this program, have shown massive difference in teaching efficacy and reported lower level of anxiety ( Jennings *et al*., 2019). Studies by ( Jennings *et al*., 2013, 2019; Telles *et al*., 2018; Rahm & Heise, 2019; Dyer *et al*., 2020; Hepburn *et al*., 2021a) implemented CALM program. It included sessions on yoga, relaxation, breathing exercises, gratitude, loving and caring, self-care and shared experience with colleagues. The results of CALM and SMART program showed significant increase or improvement in work related functioning, better adaptability towards work climate, reduction in emotional exhaustion ( Roeser *et al*., 2012; Jennings *et al*., 2013). Recently investigators have introduced innovative mindfulness techniques such as emotive behaviour theory, inquiry-based stress reduction and mentalising programs. It was observed that these interventions manifested positive improvements in productivity and wellness of controlled group participants.

Through review it was evident that relational and intrapersonal stressors were second contributors for the persistent stress level among teachers. Coping mechanism (14 studies) such as social support (12 studies) and improved managerial (8 studies) consisting of individual and organisational interventions were designed to overcome the above-mentioned stress factors. The interventions were delivered in the form of seminars, training session on stress awareness, stress management tactics, conflict management, school enculturation technique, cognitive and behavioural coping ( Bermejo-Toro *et al*., 2015; Prilleltensky *et al*., 2016; Aulén *et al*., 2021), workplace change ( Carroll *et al*., 2020). That resulted in meeting the job demands, having a work life balance and improved student teacher relationship, meeting the expectations of management, colleagues and parents, increase in self efficacy and improved work engagement.

### Limitations and future research agenda

The current scoping review have certain limitations. At the first instance, the articles are published in English and only three available databases were considered for the study, which resulted in loss of data reported in other languages or the studies from other databases. Secondly, articles that examined stress factors in alliance with psychological well-being were only considered, hence further researchers can explore the association of stress with occupational well-being. Thirdly, this review was limited to only schoolteachers from primary and secondary level school, kindergarten and higher education teachers can be the future scope to study and analyse the stress factors and the interventions.

Though it can be observed, researchers have adopted diverse stress management interventions to cope with the stress and enhance well-being. In summary it can be concluded all the strategies aimed at attaining balance to work life and promote overall growth and development of teachers professionally and personally. Yoga intervention programs were adopted as a mechanism to overcome stress. For instance, Harris *et al*. (2015) conducted sessions on gentle yoga for educators which included meditation practices. Another group of researchers implemented interventions through residential programs which mainly comprised of bhakti yoga (mediation on Sanskrit syllable), Surya namaskar (sun salutation), Sukshma vyayama (relaxation exercise), shavasana (guided relaxation), pranayama (voluntarily regulated breathing, aasanas (posture) and meditation ( Telles *et al*., 2018; Dyer *et al*., 2020) designed a 3-days program comprising of physical exercises and yoga postures, sitting meditation and breathing exercises ( Hepburn *et al.*, 2021b) introduced meditation practices (samyama), ethical principles (yama niyama), posture asana (asanas). All these yoga interventions focused on eliminating personal stress. Therefore, future researchers can evaluate the effectiveness of these intervention at an organisational level. There were relatively lower studies focussing on the systematic stress factors. Hence, future research can focus on exploring the impact of policy related changes on the well-being of teachers. Additionally, a larger emphasis can be placed on a qualitative approach to better understand the fundamental problems relating to the workplace, work climate, and work culture. Lastly, it was observed that the definition of psychological well-being has been operationalized according to the context of the study by different authors, therefore, future research can explore in framing a common definition through a meta-analysis considering heterogeneity.

## Conclusion

This scoping review aimed at identifying the stress factors and the interventions/measures followed to overcome the stress among teaching profession. The stress management techniques have identified numerous health benefits when the teachers become able to handle stressful situations. Programs using different approaches are required to help the teaching professionals to deal with different stress factors inherent in their cognitively challenging and physically demanding work. A successful intervention must address the genuine issues such as the social, emotional, or physical challenges that teachers are experiencing and assist them in coping with their demanding circumstances. The study outcome helps the school management to ensure psychological well-being through interventions. The higher the psychological well-being results in overcoming depression, poor performance, poor performance, poor teacher student relationship, lack of self-confidence and improved quality in the classroom. Additionally, this study helps managers working in consultancies and organizations involved in manpower training for education industry to think and apply intervention to attain better harmony of our real drivers of the education sector.

## Data Availability

All data underlying the results are available as part of the article and no additional source data are required. Figshare. Annexure A final.doc DOI: 10.6084/m9.figshare.22324336 This project contains the following underlying data: Tabular representation of the included studies for the review Figshare. Supplementary file - Figures.docx DOI: 10.6084/m9.figshare.22324549 This project contains the following underlying data:
• Supplementary file – Figures • Prisma check list Data are available under the terms of the Creative Commons Zero “No rights reserved” data waiver (CC0 1.0 Public domain dedication).
