# Supplementary material for: Prevalence of stress and its relevance on psychological well-being of the teaching profession:  A scoping review
Source: F1000Res. 2024 Jan 15;12:424. Originally published 2023 Apr 20. [Version 2] doi: 10.12688/f1000research.131894.2 (PMC11043664; doi:10.12688/f1000research.131894.2)
Supplement: Peer review history

# Reviewer report: Reviewer response for version 2

**DOI:** 10.5256/f1000research.160190.r242854

**Published:** 2024-03-06

**Authors:** Dzintra Ilisko

**Affiliations:** 1 Daugavpils University, Daugavpils, Latvia

This scoping review has identified the stress factors and the interventions  to overcome the stress among teaching profession. The  scoping review reveals different stressors which impact psychological well-being of teachers. The authors summarize the most used interventions to overcome stress among teachers. The content succinctly described and contextualized with respect to previous and present theoretical background. The arguments and discussion of findings coherent, balanced and compelling.The conclusions partly. supported by the results presented in the article.

 The authors have written scoping review in line with PRISMA guidelines and protocol by clear indication of inclusion criteria while screening  relevant and eligible  articles. The authors adopted technique for synthesizing literature evidence which gave a broader perspective and the extent of the research activity in terms of the topic, literature gaps. The paper provides  future direction to the researchers in regards to teachers' psychological well being. The authors refer to Bronfenbrenner’s ecological systems framework (Bronfenbrenner, 1992). as an effective framework that captures a wide range of stress factors ranging from micro and macro systems. Limitations have also been indicated by the authors of the study. Stull definition of well being is required. There is no information on the date the search was carried out and indication that it was done according to PRISMA protocol.

Is the review written in accessible language?

Yes

Are all factual statements correct and adequately supported by citations?

Yes

Are the conclusions drawn appropriate in the context of the current research literature?

Yes

Is the topic of the review discussed comprehensively in the context of the current literature?

Yes

Reviewer Expertise:

Education, teachers' psychological stressors, sustainability competencies.

I confirm that I have read this submission and believe that I have an appropriate level of expertise to confirm that it is of an acceptable scientific standard.

# Reviewer report: Reviewer response for version 1

**DOI:** 10.5256/f1000research.144780.r226814

**Published:** 2024-04-24

**Authors:** Moh'd Shoqeirat

**Affiliations:** 1 Department of Psychology, Faculty of Art and Science, Al-Ahliyya Amman University, Amman, Amman Governorate, Jordan

Review for the paper:

 Prevalence of stress and its relevance on the psychological well-being of the teaching profession:  A scoping review

  

- The review covers the period between 2010 to 2022.
- Although the title implies all the teaching professions, the study sample includes only school teachers.
- The study scope is well-defined, identifying the stress factors and the interventions/measures followed to overcome the stress in the teaching profession.
- The criteria for selecting the sample and the exclusion was well defined.
- I believe the topic of the study is significant as it takes the school teachers as a sample, and the procedures used are suitable for this kind of study.
- The statistics and layout of the results clarify the scope of the study.
- The discussion includes increased research in the field (from three countries), theoretical perspective and stress intervention.
- The authors clearly clarify the limitations of the study.
- The conclusion: The paper identifies the benefit of higher psychological well-being of the teachers' profession. Also, they stress that the study results help managers working in consultancies and organisations think and apply the intervention better to harmonise our real drivers of the education sector.
- It seems that the English language may need revision.
- Finally, this paper is critical and worth for approving, as it touches on an essential societal profession. The scientific procedure followed in this study is well-defined and appropriate for such a study.

Is the review written in accessible language?

Yes

Are all factual statements correct and adequately supported by citations?

Yes

Are the conclusions drawn appropriate in the context of the current research literature?

Yes

Is the topic of the review discussed comprehensively in the context of the current literature?

Yes

Reviewer Expertise:

-         this paper is critical and worth publishing, as it touches on an essential societal profession. The scientific procedure followed in this study is well-defined and appropriate for such a study.

I confirm that I have read this submission and believe that I have an appropriate level of expertise to confirm that it is of an acceptable scientific standard.

# Reviewer report: Reviewer response for version 1

**DOI:** 10.5256/f1000research.144780.r205242

**Published:** 2023-10-05

**Authors:** Ana Patricia Ayala

**Affiliations:** 1 University of Toronto, Toronto, Canada

Thank you for the opportunity of reviewing this article.

 My observations focus in two main areas: The clarity of the study regarding grammar, and syntax, and in the methodological adherence, and transparency of the review.

**Introduction**

- I suggest adding citations to support the statements regarding teachers' effectiveness.
- Define well-being. This is a broad term and includes numerous aspects that contribute to it.
- Please clarify how developing organizational understanding is linked to bestowing this responsibility to the educational institution. This also a broad term. What does this imply? High schools? Universities?

**Methods**

- Can you provide a link to the protocol for this scoping review? Was a protocol developed? All scoping reviews are protocol driven reviews, and an a priori protocol is necessary to mitigate bias.
- Can you please indicate what reporting guideline you've used to report the study? I don't see mention of the authors' using PRISMA-SCr, the reporting guideline for Scoping reviews.

**Research questions**

- It would be extremely helpful if authors kindly clarified what is meant by item a. "What are the descriptive findings of the selected studies?" 
  What does descriptive findings mean? 

**Search strategy**

- Can authors please provide the full search strategies for all databases searched exactly as they were ran? I'm afraid the way that the search has been described is not reproducible or transparent. From the information that is supplied, the search does not seem comprehensive enough to have effectively captured all the information regarding this topic.
  There is no information regarding the date the search was ran, or updated, if it was updated at all. What platforms were used. Scopus and Web of Science are not databases, they contain many databases and different collections.
- I strongly suggest authors use PRISMA-Search to report the findings of their search, as well as their documentation of the search process.
  The sources of information are not sufficient to capture the topic at hand. Sources like APA PsycINFO, EBSCO CINAHL, and education focused databases should've been searched as well.
  The exclusion of grey literature should be addressed in the limitations of the study

**Inclusion and exclusion criteria**

 Kindly explain why the authors only looked at that date range? What is the justification to only include articles from these years?

 This should be included as a limitation to the study, and the steps the authors took to ameliorate it.

 Could authors kindly indicate how the first and second level of screening were conducted? In the description under inclusion decision based on inclusion and exclusion criteria, the authors don't explicitly state how they performed each level of screening, and they must do so for transparency purposes. 

 I also recommend they change the title to "Study selection process" to more accurately reflect the actions taken, and in accordance with language used in the methodologies the authors' cite for conduct.

**Charting the data**

 Please describe how the data extraction form was piloted

Is the review written in accessible language?

Partly

Are all factual statements correct and adequately supported by citations?

Partly

Are the conclusions drawn appropriate in the context of the current research literature?

Partly

Is the topic of the review discussed comprehensively in the context of the current literature?

No

Reviewer Expertise:

My area of research include knowledge syntheses, transparency in reporting, development of reporting guidelines

I confirm that I have read this submission and believe that I have an appropriate level of expertise to state that I do not consider it to be of an acceptable scientific standard, for reasons outlined above.

## References

[1] : PRISMA-S: an extension to the PRISMA Statement for Reporting Literature Searches in Systematic Reviews. *Syst Rev* .2021;10(1) : 10.1186/s13643-020-01542-z 39 10.1186/s13643-020-01542-z 33499930 PMC7839230

[2] : PRISMA Extension for Scoping Reviews (PRISMA-ScR): Checklist and Explanation. *Ann Intern Med* .2018;169(7) : 10.7326/M18-0850 467-473 10.7326/M18-0850 30178033
